# A Case of Ectopic Ureter With Right Atrophied Kidney in a Pediatric Patient

**DOI:** 10.7759/cureus.53360

**Published:** 2024-02-01

**Authors:** Divyanshi Kaplish, Jayant D Vagha, Revat J Meshram, Sham Lohiya, Priyanka Hampe

**Affiliations:** 1 Pediatrics, Jawaharlal Nehru Medical College, Datta Meghe Institute of Higher Education and Research, Wardha, IND

**Keywords:** urinary bladder, reimplantation, renal atrophy, atrophy, ureter

## Abstract

An ectopic ureter (EU) is a ureter that does not connect appropriately to the bladder and drains somewhere other than the urinary bladder. Ectopic ureter is not so common in kidney anomalies. In men, the EU usually opens near the prostate into the urethra; however, in females, it mainly opens into organs of reproduction or into the urethra. Differential diagnosis of urinary incontinence from other causes, such as EU has a potential cure through surgery. Most women with ectopic ureters have duplex kidneys. An EU emptying a single-system ectopic dysplastic but functioning kidney is uncommon, especially in females. Computed tomography and magnetic resonance imaging provide a clearer image of the ectopic kidney. The surgical techniques used to correct this type of EU are determined based on the functioning of the kidney and anomalies related to the EU site. This is a case of a 9-year-old female who presented with complaints of dribbling urine, which was discovered to be caused by an ectopic ureter with an atrophic kidney.

## Introduction

An ectopic ureter (EU) is a ureter opening other than the trigone of the bladder. Leakage of urine continuously and frequent intentional urination can be an indication of the presence of an ectopic ureter, especially in females. When an ectopic ureter drains only one kidney, it is referred to as a single-system ectopic ureter (SSEU) [1]. Functional issues frequently cause incontinence of urine in children, and management is frequently ineffective as a result. However, incontinence caused by EU is especially important as it is potentially curable through surgical repair. Ureteral duplication accounts for 80% of all EU cases in women [2]. SSEU with dysplastic kidneys is, however, not so common in females [3]. The surgical approaches used to treat SSEU are influenced by renal function extent, accompanying anomalies, and EU site. Nephroureterectomy is indicated when a dysplastic kidney has limited function while the contralateral kidney is normal. When kidney function is preserved, ureteric reimplantation is the preferred procedure for restoring continence and sparing the function of the remaining nephrons [4]. This case report will help the healthcare professional to deal with cases of EU, by proper history taking, timely diagnosis, and treatment. 

## Case presentation

**Figure 1:**
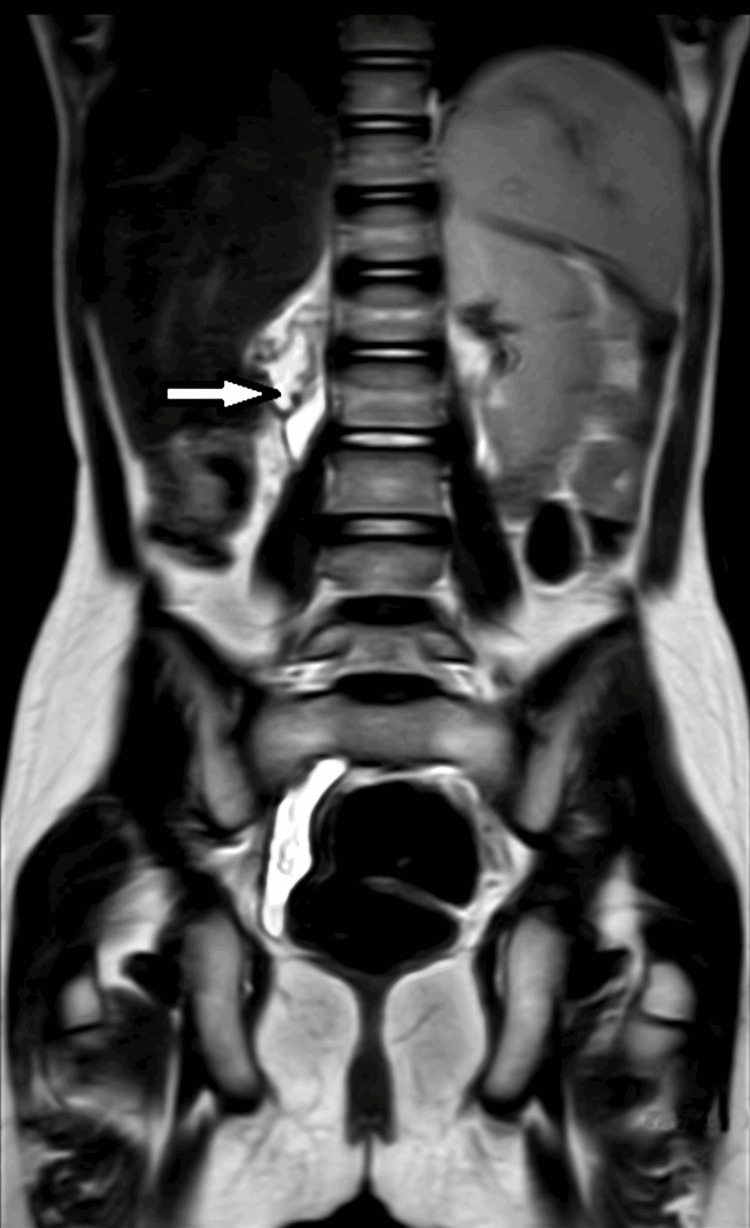
MRI showing atrophied right kidney (2.8 × 1 cm)

**Figure 2:**
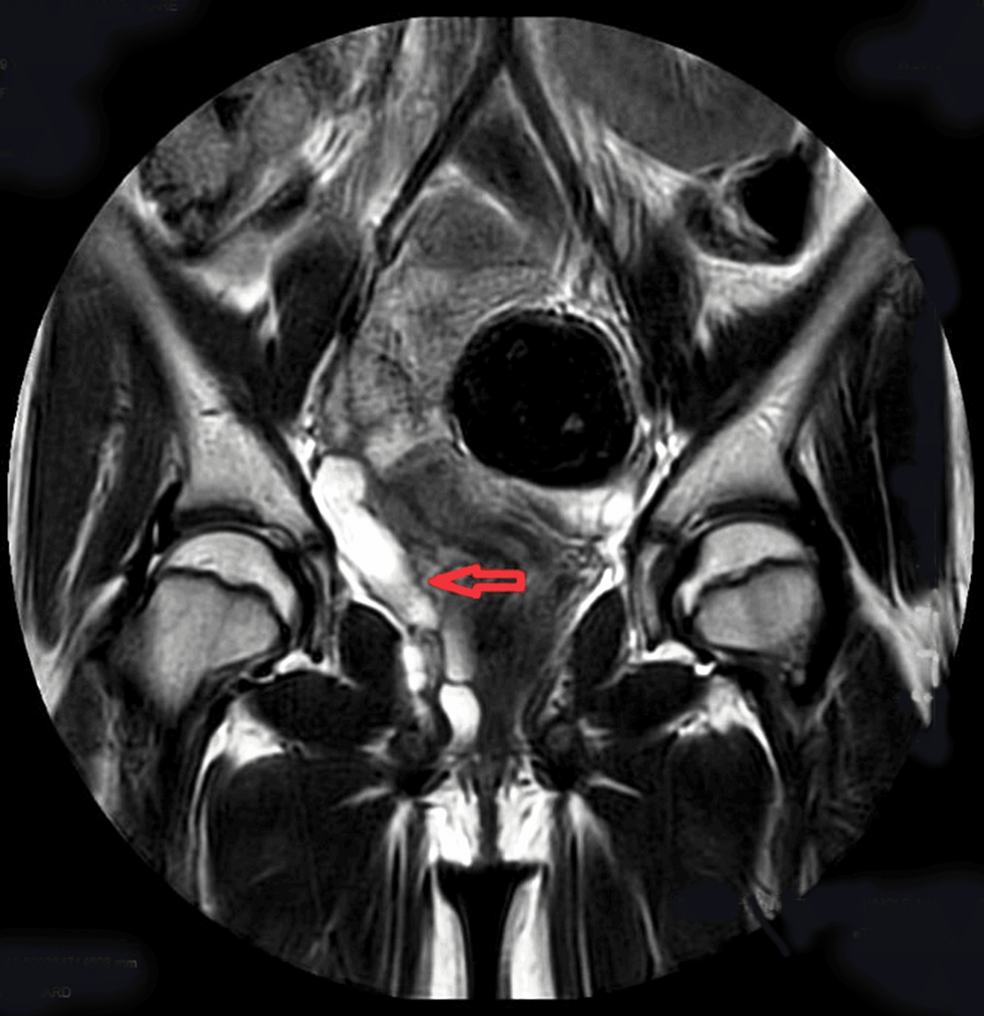
MRI showing moderate to gross right-sided hydroureter; it is tortuous in course with ectopic insertion in urethra near external urethral orifice

A 9-year-old female child presented with a complaint of dribbling urine along with a normal voiding pattern since birth. She had complaints of persistent dampness, with or without occasional regular urination from birth, along with varying levels of abdominal discomfort and occurrences of urinary tract infections. There was no urgency, frequency, or dysuria. On admission, the patient had normal pulse pressure, Respiratory rate of 24/min, BP of 110/80 mm hg, and SpO_2_ of 98% on room air. Abdominal examination revealed a soft abdomen, non-tender, and non-palpable liver and spleen. Blood investigations suggested a hemoglobin level of 8.6 gm/dl, total leucocyte count (TLC) of 16900/cumm, platelets of 410000 per microliter, and hematocrit of 27.5%. A urine routine examination revealed nil urine albumin, nil urine sugar, 2-3 pus cells/high power field, and 2-3 epithelial cells/high power field. Ultrasonography of the abdomen/pelvis revealed an atrophic right kidney (small) with query hydrosalpinx (dilated tortuous tubular structure notes in right adnexa). MRI was advised, which was suggestive of an atretic right kidney (2.8 × 1 cm) (Figure 1), with ectopic insertion of the hydropic ureter in the urethra (Figure 2).

**Figure 3:**
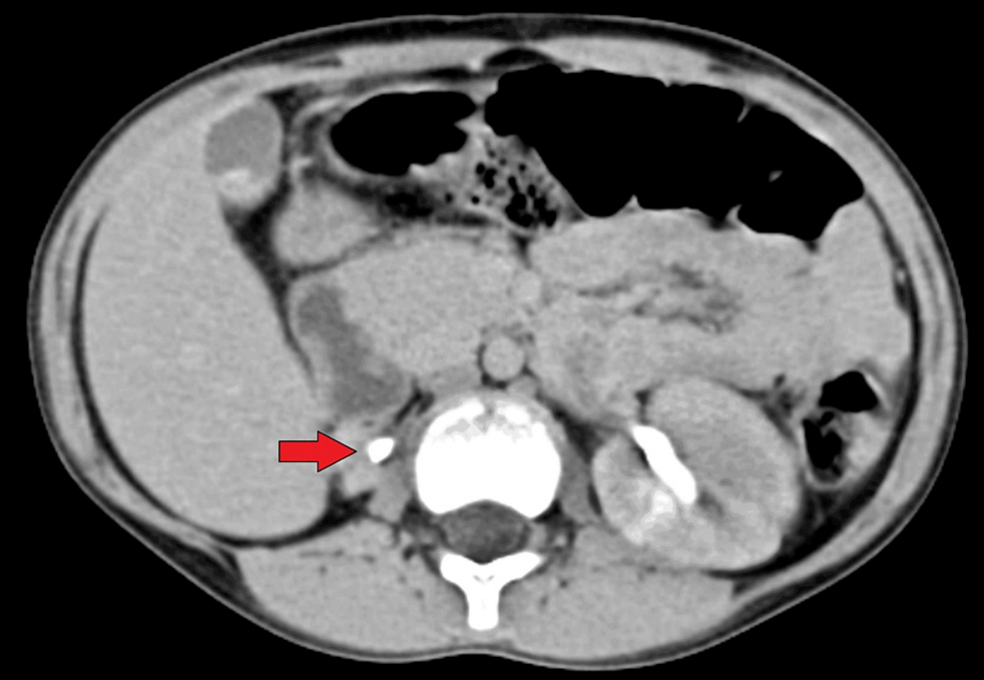
CT urography showing atrophic right kidney. Gross ureter dilatation throughout the course with proximal ureteric diameter 1.6 cm, mid-ureteric diameter 1.7 cm, and distal ureteric diameter 2.4 cm, suggestive of hydroureter

The normal left kidney and left ureter are well visualized up to the crossing of iliac vessels. Cystoscopy examination revealed the left ureteric orifice. The right ureteric orifice is not visualized. Ectopic ureteric orifice visualized between urethra and vagina. The orifice is stenotic, with continuous urine dribbling seen. The bladder appeared to be normal. CT urography revealed a right ectopic ureter with a gross hydroureter; ectopic insertion of right tortuous ureter noted in the urethra near the external orifice at the vaginal vestibule; right atrophic kidney; cholelithiasis (Figure 3). No vesicoureteric reflux was noted preoperatively, so micturating cystourethrogram was not done.

The patient was advised diethylenetriaminepentaacetic acid (DTPA) renal scan which revealed a 20% functioning right kidney, and a total glomerular filtration rate of 45.6 ml/min. The patient was planned for kidney preservation ureter reimplantation, the Lich-Gregoir technique was used, and Double-J (DJ) stent was placed successfully with an operative time of 2 hrs 20 mins, 50 ml blood loss, done under general anesthesia. The DJ stent was removed after 2 weeks. At the three-month follow-up, intravenous urography revealed no obstruction, and a micturating cystourethrogram revealed no reflux the patient was continent and is now on regular follow-up, and nephrectomy is planned for right atrophic kidney after 6 months of follow up as patient was not ready for nephrectomy at that time and reimplantation was done to alleviate the symptom of dribbling of urine or urinary incontinence.

## Discussion

The prevalence of EU is unknown because most EU patients are asymptomatic and the diagnosis is quite often missed. According to the literature, SSEU is common in males. Gangopadhyaya et al. found SSEU in females. In our case also, an SSEU was present. Pelvic MRI is the preferred mode of diagnosis. Ureteral duplication is responsible for 80% of ectopic ureter cases in women. An EU can be caused by any underlying condition in common nephric duct apoptosis or the location of ureteric bud origin. If the ureteric bud emerges more cephalad than normal, the ectopic ureter may be inserted distally [5]. Urinary drainage into the female reproductive tract may occur if the ectopic ureteric bud becomes incorporated into the paramesonephric duct derivatives. In females, the ectopic ureteric opening may be located anywhere from the bladder neck to the perineum, with the urethra, vagina, and vestibule being the common sites of entry [6,7]. As a result, in most females, Incontinence occurs when the EU drains either distal to the urethral sphincter or into the reproductive tract. It has also been reported that the EU drains into the rectum. Our patient has the opening of the urethra into the vaginal orifice [8]. A case of SSEU was reported by Prakash et al. draining into Gartner's cyst, which was then managed by ureteral reimplantation laparoscopically [9].

The results of a clinical examination are highly non-specific. The clinical findings may include urine pooling at the introitus, Gartner's cyst, etc. The mainstay for diagnosing EU is USG, CT urography, dimercaptosuccinic acid (DMSA) scan, and voiding cystourethrography (VCUG) [10].

The surgical treatment of an SSEU is determined by the degree of renal function, associated anomalies, and the location of the EU [11]. In SSEU, the kidney is usually smaller and underdeveloped; the treatment is nephroterectomy. If the function of the kidney is normal and the kidney is not dysplastic, the location of the EU is the most critical factor influencing the surgery. Ureteral reimplantation is used to repair an extramural ectopic ureter. Surgery aims to relocate the ureteral orifice closer to the urethral sphincter, restoring urinary outflow control. Grover et al. described a case of the right EU terminating in the right lateral wall of the vagina, with good right kidney function managed by right ureteric re-implantation [12,13]. Treatment for patients with ectopic ureters and dysplastic kidneys aims to alleviate symptoms as soon as possible.

## Conclusions

This is the case of a 9-year-old female patient with an ectopic ureter and non-functional, atretic right kidney which was successfully treated with reimplantation. This case further adds to the body of knowledge that urinary incontinence in children after toilet training should be thoroughly evaluated for ectopic ureter; additionally, single-system ectopic ureter is uncommon. Urine incontinence that persists after toilet training necessitates a thorough assessment so that appropriate treatment can be instituted in childhood while not meddling with psychological development. In the case of urinary incontinence, this case suggests that, particularly in females, congenital genitourinary tract anomalies should be considered. The surgical management is determined by the extent of renal function and ectopic ureter location. Incontinence can be treated with nephroureterectomy or ureteric reimplantation.
